# Complexity-Entropy Causality Plane as a Complexity Measure for Two-Dimensional Patterns

**DOI:** 10.1371/journal.pone.0040689

**Published:** 2012-08-14

**Authors:** Haroldo V. Ribeiro, Luciano Zunino, Ervin K. Lenzi, Perseu A. Santoro, Renio S. Mendes

**Affiliations:** 1 Departamento de Física and National Institute of Science and Technology for Complex Systems, Universidade Estadual de Maringá, Maringá, Brazil; 2 Centro de Investigaciones Ópticas (CONICET La Plata - CIC), C.C. 3, Gonnet, Argentina; 3 Departamento de Ciencias Básicas, Facultad de Ingeniería, Universidad Nacional de La Plata, La Plata, Argentina; Northwestern University, United States of America

## Abstract

Complexity measures are essential to understand complex systems and there are numerous definitions to analyze one-dimensional data. However, extensions of these approaches to two or higher-dimensional data, such as images, are much less common. Here, we reduce this gap by applying the ideas of the permutation entropy combined with a relative entropic index. We build up a numerical procedure that can be easily implemented to evaluate the complexity of two or higher-dimensional patterns. We work out this method in different scenarios where numerical experiments and empirical data were taken into account. Specifically, we have applied the method to 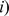 fractal landscapes generated numerically where we compare our measures with the Hurst exponent; 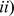 liquid crystal textures where nematic-isotropic-nematic phase transitions were properly identified; 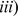 12 characteristic textures of liquid crystals where the different values show that the method can distinguish different phases; 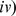 and Ising surfaces where our method identified the critical temperature and also proved to be stable.

## Introduction

Investigations related to the so called complex systems are widely spread among different scientific communities, ranging from physics and biology to economy and psychology. A considerable part of these works deals with empirical data aiming to extract patterns, regularities or laws that rule the dynamics of the system. In this direction, the concept of complexity measures often emerges. Complexity measures can compare empirical data such as time series and classify them in somewhere between regular, chaotic or random [1], while other complexity measures can differentiate between degrees of correlations [2]. Examples of these measures include algorithmic complexity [3], entropies [4], relative entropies [5], fractal dimensions [6], and Lyapunov exponents [7]. These seminal works are still motivating new definitions, and today there are numerous definitions of complexity, which have been successful applied to different areas such as medicine [8], [9], ecology [10]–[13], astrophysics [14]–[16], and music [17], [18].

It is surprising that this large number of complexity measures is mainly focused on one-dimensional data, while much less attention has been paid to two and higher-dimensional structures such as images. Naturally, there are few exceptions such as the work of Grassberger [19] and more recent Refs. [20]–[22], though some of the authors of these papers agree that a higher-dimensional approach still represents an open and subtle problem. Furthermore, as it was stated by Bandt and Pompe [23], most of the complexity measures depend on specific algorithms or recipes for processing the data which may also depend on tuning parameters. As a direct consequence, there are huge difficulties for reproducing previous results without the knowledge of details of the methods.

Bandt and Pompe not only raised this problem, but they also proposed an alternative method that tries to overcome the previous problems, introducing what they call *permutation entropy* – a *natural* complexity measure for time series. There are many recent applications of this new technique that confirm its usefulness [24]–[31]. In particular, Rosso et al. [1] have successful applied the Bandt and Pompe ideas together with a relative entropic measure [32] to differentiate chaotic time series from stochastic ones. They have constructed a diagram, which was first proposed by López-Ruiz et al. [33], (called as complexity-entropy causality plane) by plotting the relative entropic measure versus the permutation entropy. Intriguingly, chaotic and stochastic series are located in different regions of this representation space.

Here, we show that the complexity-entropy causality plane can be extended for higher-dimensional patterns. We apply this new approach in different scenarios related to two-dimensional structures and the results indicate that the method is very promising for distinguishing between two-dimensional patterns. The following sections are organized as follows. Section II is devoted to review briefly the properties of the permutation information-theory-derived quantifiers and the complexity-entropy causality plane, and also to define an appropriate way to generalize these definitions to higher-dimensional data. In Section III, we work out several applications based on numerical and empirical data. Section IV presents a summary of our results.

## Methods

The ingenious idea of Bandt and Pompe [23] was to define a measure that may be easily applied to any type of time series. The method lies on associating symbolic sequences to the segments of the time series based on the existence of local order, and next, by using probability distribution associated to these symbols, to estimate the complexity quantifier. For purpose of definition, let us consider a time series 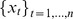 composed by 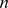 elements and also 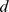-dimensional vectors (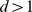) defined by
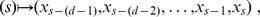
where 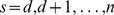. Next, for all the 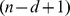 vectors, we evaluate the permutations 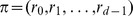 of 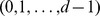 defined by 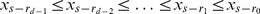. The 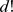 possible permutations of 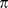 will be the accessible states of the system, and for each state we estimate the ordinal pattern probability given by



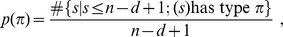
where the symbol # stands for the number of occurrences of the permutation 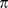. Now, we can apply the ordinal patterns probability distribution, 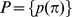, to estimate a complexity measure based on some entropic formulation.

Before advancing, we note that the previous method may be extended to higher-dimensional data structures such as images. In order to do this, we consider that the system is now represented by a two-dimensional array 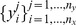 of size 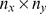. In analogy to the vector 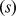, we define 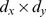 matrices (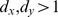) given by
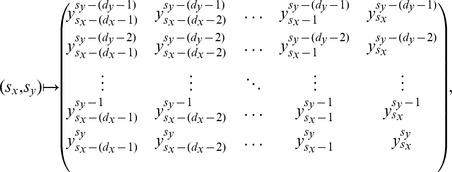
where 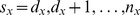 and 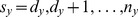. Next, for all these 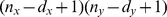 matrices, we evaluate the permutations 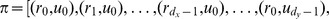 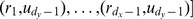 of 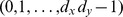 defined by 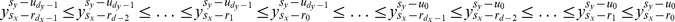. The system can now access 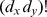 states for which we calculate the probability distribution 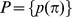 through the relative frequencies given by



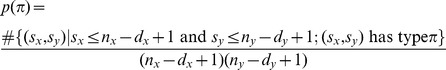



**Figure 1:**
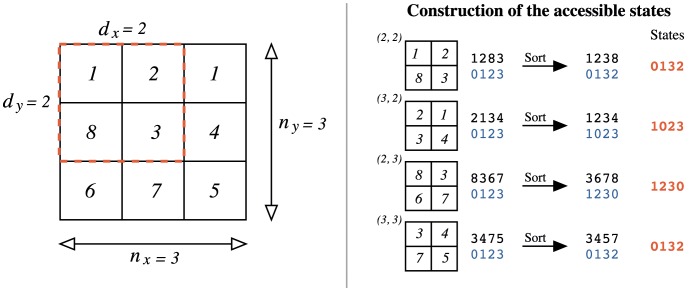
Schematic representation of the construction of the accessible states. In this example we have a 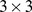 array (left panel) and we choose the embedding dimensions 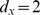 and 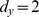. In the right panel we illustrate the construction of the states. We first obtain the sub-matrix corresponding to 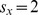 and 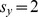 that have as elements 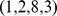 and, after sorting, this sub-matrix leads to the state “0132”. We thus move to next sub-matrix 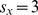 and 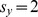 which have the elements 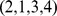 and that, after sorting, leads to the state “1023”. The last two remaining matrices lead to the states “1230” and “0132”. Finally, we estimate the probabilities 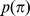, that are, 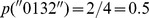, 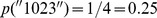 and 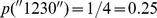 which are then used in the equations (1) and (2), leading to 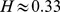 and 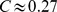.

For easier understanding, we illustrate this procedure for a small array in Fig. 1.

Naturally, the order procedure that defines the permutation 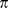 is no longer unique as in the one-dimensional case. For instance, instead of ordering the elements of 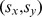 row-by-row, we could also order column-by-column. However, these other definitions will only change the “name” of the states in such a way that the set 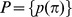 will remain unchanged. Thus, there is no lost of generalization in assuming a given order recipe for defining 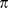.

We note that this procedure is straightforward generalized to accomplish higher-dimensional structures (e.g., the volumetric brain images obtained via functional magnetic resonance imaging), and that it recovers the one-dimensional case by setting 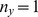 and 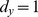. Here, for simplicity, we focus our analysis on two-dimensional structures.

The parameters 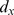 and 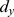 (known as embedding dimensions) play an important role in the estimation of the permutation probability distribution 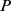, since they determine the number of accessible states. In the one-dimensional case, it is usual to choose 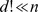 in order to obtain reliable statistics in the one-dimensional case (for practical purposes, Bandt and Pompe recommend 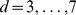 [23]). For the two-dimensional case a similar relationship must hold, i.e., 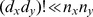. To go further, we need to rewrite the entropic measures used in Refs. [1], [23]. The first one is called normalized permutation entropy [23] and it is obtained by applying the Shannon’s entropy to the probabilities 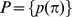, i.e.,
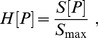
(1)where 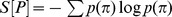 and 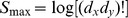. The value of 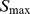 is obtained by considering all the 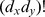 accessible states to be equiprobable, i.e., 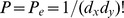. By definition, 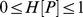, where the upper bound occurs for a completely random array. We expect 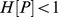 for arrays that exhibit some kind of correlated dynamics.

The other measure [1] is defined by.
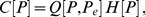
(2)where 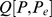 is a relative entropic metric between the empirical ordinal probability 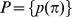 and the equiprobable state 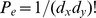. The quantity 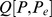 is known as disequilibrium and it is defined in terms of the Jensen-Shannon divergence [34] (or also in terms of a symmetrized Kullback-Leibler divergence [35]) and can be written as
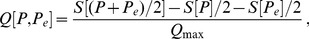
(3)where



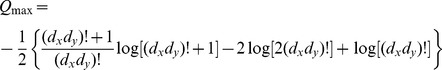
is the maximum possible value of 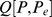, obtained when one of the components of 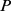 is equal to one and all the other vanish.

The disequilibrium 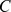 quantifies the degree of correlational structures providing important additional information that may not be carried only by the permutation entropy. In addition, for a given 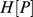 value there exists a range of possible values for 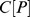 [36]. This is the main reason why Rosso et al. [1] proposed to employ a diagram of 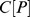 versus 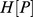 as a diagnostic tool, building up the complexity-entropy causality plane.

## Results and Discussion

In the following, we will calculate the diagram of 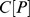 versus 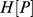 to measure the complexity and to distinguish among different two-dimensional patterns.

### Fractal Surfaces

**Figure 2:**
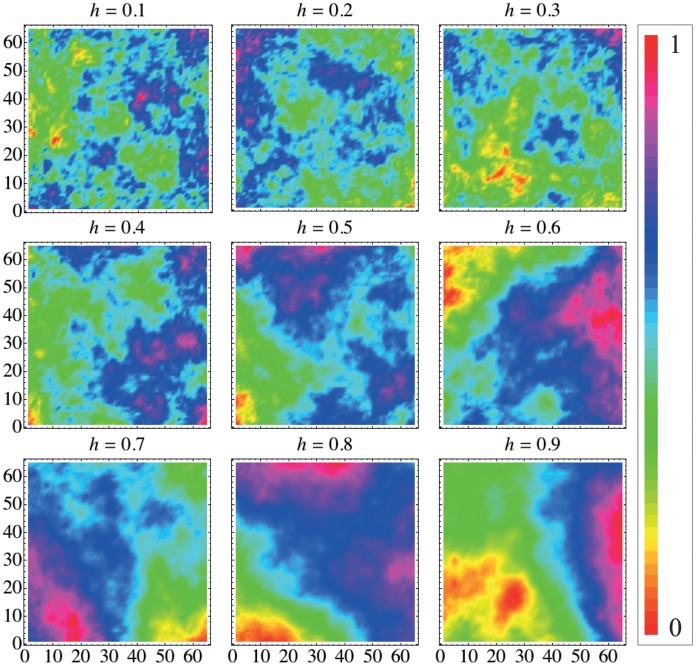
Examples of fractal surfaces obtained through the random midpoint displacement method. These are 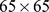 surfaces (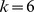) for different values of the Hurst exponent 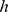. For easier visualization, we have scaled the height of the surfaces in order to stay between 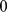 and 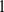. We note that for small values of 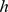 the surfaces display an alternation of peaks and valleys (anti-persistent behavior) much more frequent than those one obtained for larger values of 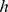. For larger values of 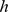, the surfaces are smoother reflecting the persistent behavior induced by the value of 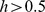.

We generate fractal surfaces through the random midpoint displacement algorithm [37]. This algorithm starts with a square. For each vertex, we assign a random value representing the surface height. Next, we add a new point located at the center of the initial square. We set the height of this point equal to the average height of the previous four vertex plus a Gaussian random number with zero mean and standard-deviation 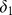. We also add four points located at the middle segments which connects each initial vertex. For these four points, the heights are equal to the average value between the two closest vertex and the middle point plus a Gaussian random number with zero mean and standard-deviation 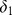. Now, we imagine that these 9 points represent four new squares and, for each one, we apply the previous procedure using 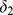. By repeating this process 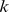 times and using 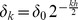, we should obtain a square surface of side 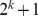 with fractal properties. Here, 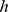 is the Hurst exponent and 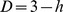 is the surface fractal dimension. Figure 2 shows several surfaces generated through this procedure for different values of 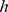.

**Figure 3:**
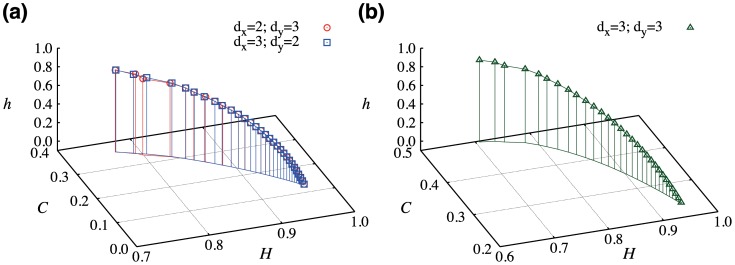
Dependence of the complexity-entropy causality plane on Hurst exponent *h* . We have employed fractal surfaces of size 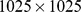 (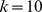). In (a) we plot 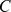 and 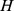 versus 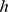 for the embedding dimensions 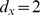 and 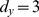 (circles) and also for 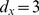 and 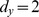 (squares). We note the invariance of the index against the rotation 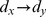 and 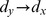. In (b) we plot the diagram for 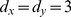. We observe changes in the scale of 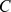 and 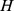 caused by the increasing number of states. In both cases, as 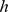 increases the complexity 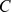 also increases while the permutation entropy 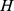 decreases. This behavior reflects the differences in the roughness shown in Fig. 2. For values of 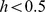 the surface is anti-persistent which generates a flatter distribution for the values of 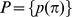 leading to values of 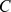 and 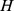 closer to the aleatory limit (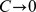 and 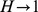). For values of 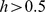 there is a persistent behavior in the surfaces heights which generates a more intricate distribution of 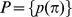 and, consequently, values of 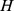 and 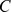 that are closer to the middle of the causality plane (region of higher complexity).

We apply our method for these surfaces aiming to verify how the permutation quantifiers 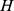 and 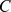 change with the Hurst exponent 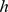, as it is shown in Fig. 3. In these 3d plots, we show the localization in the causality plane obtained for different values of 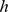 evaluated from 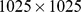 surfaces (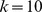). In Fig. 3a, we use 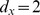 and 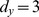 (circles), and 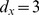 and 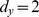 (squares) as embedding dimensions. Note that the values of 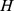 and 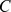 are practically invariant under the rotation 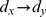 and 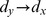. This invariance is related to the fact that in these fractal surfaces there is not preferential direction. In Fig. 3b, we employ 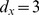 and 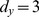. We note basically the same dependence but a different range for 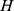 and 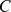, since this change increases the number of accessible states. These results show that our method properly differentiates fractal surfaces concerning the Hurst exponent. Moreover, we investigate the robustness of the permutation quantifiers under several realizations of the random midpoint displacement algorithm and the results show that both indexes are very stable. For example, the standard-deviation in the values of 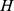 and 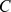 are usually smaller than 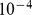 when considering 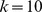.

### Liquid Crystal Textures

Another interesting application is related to different patterns that a thin film of a liquid crystal exhibits. These textures are obtained by observing a thin sample of liquid crystal placed between two crossed polarizers in a microscope. The textures give useful information about the macroscopic structure of the liquid crystal. For instance, different phases have different typical textures, and by tracking their evolution one can properly identify the phase transition.

**Figure 4:**
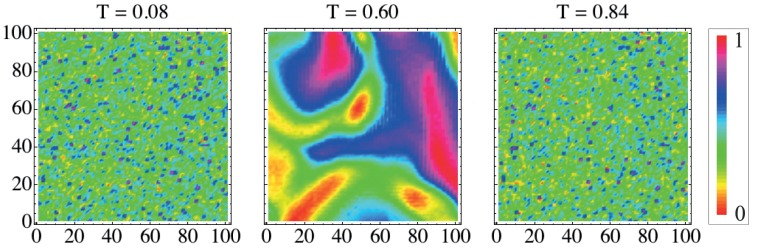
Characteristic textures of a lyotropic liquid crystal at different temperatures and phases. The lyotropic system used here is a mixture of potassium laurate 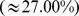, decanol 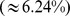 and deuterium oxide 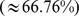 – suitable concentrations in order to get a isotropic 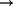 nematic 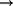 isotropic phase sequence [38]. These images were constructed by observing the optical microscopy of a flat capillary which contains the mixture at different temperatures. Here, we have used the average value of the pixels of the three layers (RGB) of the original image and a rescaled temperature.

We first study a lyotropic liquid crystal under isotropic-nematic-isotropic phase transition. Figure 4 shows three snapshots of the texture at different temperatures. In this case, we clearly note the differences in the textures. The leftmost and rightmost textures are at the isotropic phase while the middle one is at the nematic phase. We observe that the pattern is very complex for the nematic phase, while for the isotropic one it is basically random.

**Figure 5:**
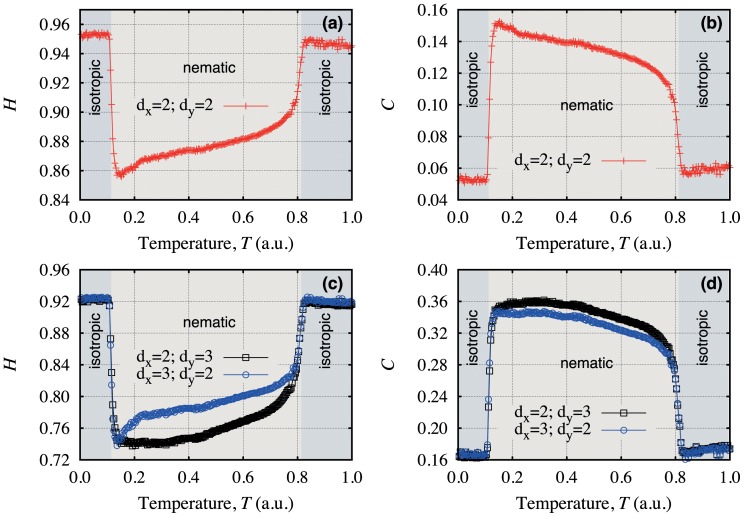
Dependence of the entropic indexes on the temperature of a lyotropic liquid crystal. We plot 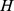 versus the temperature in (a) and 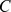 versus the temperature in (b), where we employ 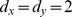. Figures (c) and (d) present the results for 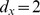 and 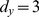, and also for 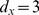 and 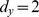. The different shaded areas represent the different liquid crystal phases. Note that the phase transitions are properly identified in all cases. Due to the asymmetry of the elongated capillary tube where the liquid crystal sample is placed, 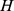 and 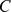 present slight differences under the rotation 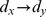 and 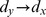.

We calculate 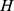 and 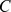 as a function of the temperature for different values of the embedding dimensions, as it is shown in Fig. 5. In these plots, the different shaded regions represent the different liquid crystal phases. We note that the phase transitions are successful identified independently of 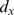 and 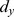. However, Fig. 5c and 5d show a slight different dependence of 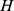 and 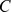 versus the temperature when considering 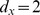 and 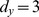 or 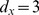 and 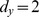. Because the liquid crystal sample is placed in elongated capillary tube, there is a surface effect that act on the liquid crystal molecules. This effect is usually amplified at the phase transition and it is also the reason for differences between the embedding dimensions.

**Figure 6:**
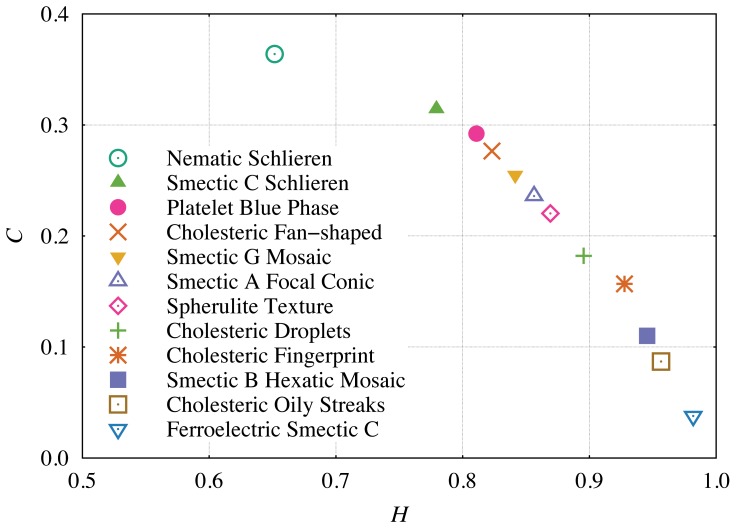
Complexity-entropy causality plane evaluated for several liquid crystal textures [**[39]**]. Here, we have used the averaged pixel values of the three layers (RGB) of the original image and 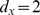 and 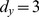. The image sizes are about 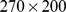 pixels. We note that each texture has a unique position in the causality plane which indicates that the permutation quantifiers are capable of differentiate not only transitions involving the isotropic phase, but also smoother phase transitions. We further observe that some high ordered phase such as the blue phase are located at the central part of the causality plane (region of higher complexity), while other phases which present a large number of defects such as the Smectic B and C are closer to the aleatory limit (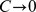 and 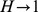).

In this particular phase transition, the difference between the textures are large enough that it can be identified just by visual inspection. However, this is not the usual case and many phase transitions are very difficult to identify. In this context, an interesting question is whether our method can help to distinguish different phases. To address this question, we evaluate 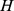 and 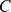 for twelve characteristic textures of different liquid crystals. We download these textures from the webpage of the Liquid Crystal Institute at Kent State University [39] and Fig. 6 shows the value of 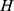 and 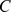 for each texture in the causality plane. The results allow to conclude that the method ranks the textures in a kind of complexity order where each characteristic texture occupies a different place in this representation space. Moreover, the different values of 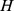 and 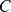 indicate that the permutation quantifiers can also identify smooth phase transitions.

Naturally, the location of each texture in the causality plane should be related to physical properties of the liquid crystals. A better understanding of the relation between the permutation quantifiers and these physical attributes may deserves a more careful investigation since some properties of liquid crystals such as the order parameter can be quite hard to empirically measure. In this context, the existence of a clear relation between, for example, the order parameter and 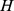 or 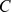 will be experimentally handy. Here, we just have the pictures of the textures in such a way that is very hard to point out these relationships. However, a visual inspection of Fig. 6 suggests that some of the more ordered phases, such as the blue phase (this phase display a cubic structure of defects), are located in the central part of the causality plane (region of higher complexity), while other textures which present a large number of non-ordered defects, such as the Smectic B and C, are positioned closer to the aleatory limit (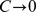 and 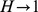). Thus, it seems that the permutation quantifiers are capturing in somehow the competition between the orientational order of the phase and, also, the number of defects present in the textures.

### Ising Surfaces

**Figure 7:**
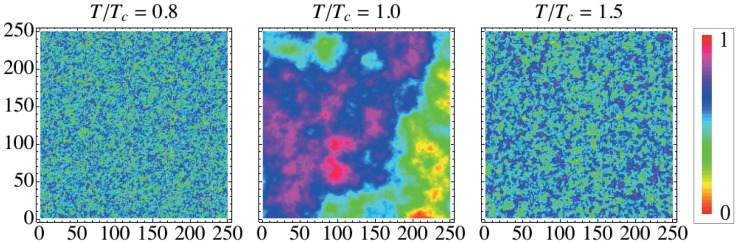
Examples of Ising surfaces for three different temperatures. These surfaces were obtained after 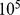 Monte Carlo steps for three different temperatures: below 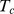, at 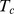 and above 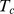. In these plots, the height values were scaled to stay between 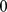 and 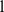. We note that for temperatures higher or lower than 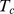, the surfaces exhibit an almost random pattern. For values of the temperature closer to 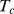 the surfaces exhibit a more complex pattern, reflecting the long-range correlations that appear among the spin sites during the phase transition.

As a last application, we study the permutation measures 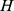 and 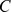 applied to Ising surfaces [40], [41]. These surfaces are obtained by accumulating the lattice spin values 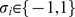 of the Ising model defined by the Hamiltonian.
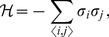
(4)where the sum is over all the pairs of first neighbor sites in the lattice. We numerically solve this spin-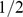 Ising model on a 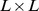 lattice using the Monte Carlo method with periodic boundary conditions. By using the spin values, we define the surface height for each lattice site 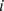 as
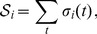
(5)where 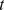 represents the number of Monte Carlo steps. In Fig. 7, we show three surfaces obtained though this procedure for different values of the reduced temperature 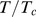, where 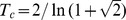 is the critical temperature of the model. We note the complex pattern exhibited by the surface for 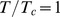, and the almost random patterns for 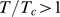 and 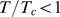.

**Figure 8:**
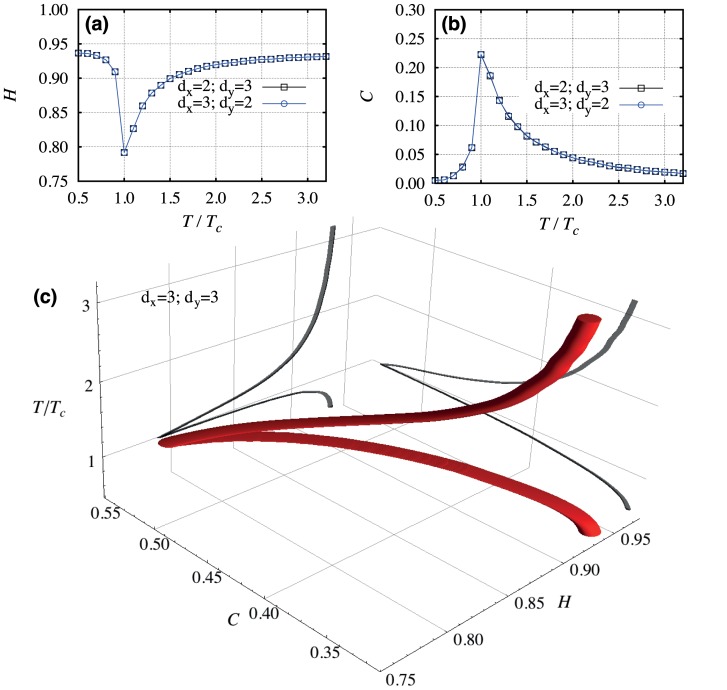
Dependence of the entropic indexes on the reduced temperature for Ising surfaces. (a) The permutation entropy 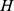 and (b) the complexity measure 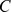 versus the reduced temperature for 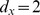 and 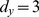, and also for 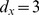 and 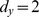. We note invariance of indexes under the rotation 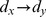 and 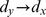. (c) A 3d visualization of the Ising model phase transition when considering 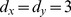. The gray shadows represent the dependences of 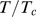 on 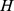 and of 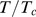 on 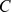.

We first investigate the dependence of 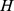 and 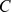 on the reduced temperature 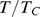 after a large number of Monte Carlo steps (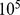) and for 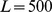. Figures 8a and 8b show 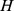 and 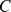 for 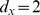 and 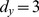, and for the rotation 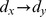 and 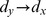. We note that, at the critical temperature, both indexes display a sharp peak and that they are invariant under the rotation. Moreover, Fig. 8c presents a 3d visualization of the phase transition for 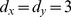. This higher-dimensional representation can be useful when investigating more complex phase transitions, since a greater number of degrees of freedom allows the critical point to be more visible.

**Figure 9:**
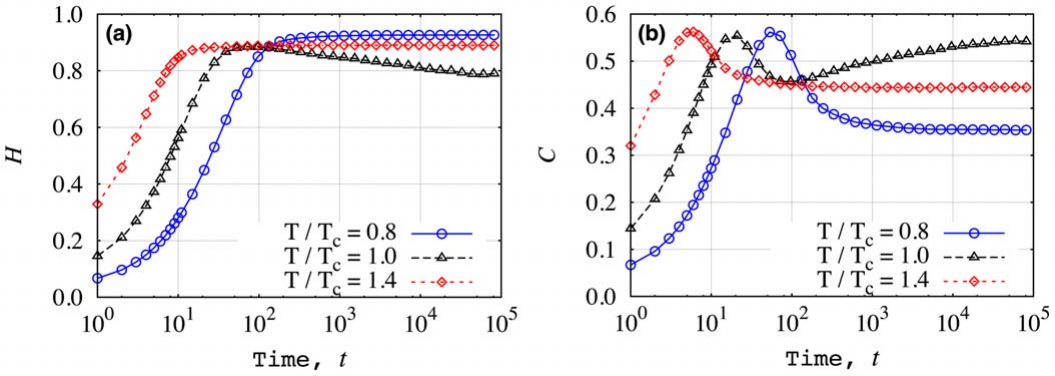
Dependence of the entropic indexes on the number of Monte Carlo steps. Here, 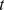 denotes the number of Monte Carlo steps and the reduced temperatures are indicated in the plots. In (a) we show 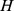 versus 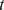 and in (b) 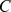 versus 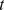 for 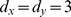. We note the stability of both indexes after 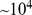 Monte Carlo steps.

We further study the temporal evolution of 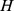 and 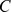 for different reduced temperatures, as it is shown in Fig. 9. The initial values of the spins were chosen equal to 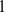 and, as we can see, the values for 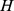 and 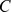 are different just after one Monte Carlo step. For 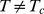, the value of 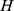 increases over time and around 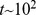 it reaches a plateau. For 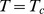, the value of 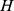 increases up to a maximum value around 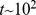 and then starts to approach a lower plateau value. A striking behavior is observed for 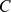, where for all temperatures the complexity displays a maximum value before it begins to approach a plateau value. It is worth noting that both quantifiers are very stable after 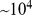 Monte Carlo steps.

### Conclusions

We have proposed a generalization of the complexity-entropy causality plane to higher-dimensional patterns. We applied this approach to fractal surfaces, liquid crystal textures and Ising surfaces. It was shown that the indexes 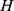 and 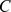 performed very well for distinguishing between the different roughness of the fractal surfaces. The indexes properly identified the phase transitions of a lyotropic liquid crystal and sorted different characteristic textures in a kind of complexity order. Finally, concerning the Ising surfaces, the indexes not only had identified the critical temperature, but also proved to be stable after 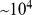 Monte Carlo steps. The method also has a very fast and simple numerical evaluation. Taking into account all these findings, we are very optimist that our method can reduce the gap between one-dimensional complexity measures and the higher-dimensional ones.
